# Beyond embryological remnants: imaging of ligamentum teres hepatis and falciform ligament pathologies

**DOI:** 10.1186/s13244-025-02116-0

**Published:** 2025-10-25

**Authors:** Sevtap Arslan, Ali Devrim Karaosmanoglu, Deniz Akata, Mustafa Nasuh Ozmen, Muşturay Karcaaltincaba

**Affiliations:** Department of Radiology, Hacettepe University School of Medicine, Sihhiye, 06230 Ankara Türkiye

**Keywords:** Liver, Falciform ligament, Ligamentum teres hepatis, Computed tomography, Magnetic resonance imaging

## Abstract

**Abstract:**

The falciform ligament and the ligamentum teres hepatis (LTH) have historically been regarded as embryological remnants with minimal clinical significance. However, their complex anatomical relationships with the liver, diaphragm, retroperitoneum, and abdominal and thoracic walls make them susceptible to various pathological conditions. This review highlights the anatomy of the falciform ligament and the LTH, their significance in surgical procedures, and pathologies of these structures on cross-sectional imaging. We will discuss the normal anatomy, congenital variations, inflammatory and infectious processes, neoplastic conditions, and vascular anomalies affecting these structures. Recognition of the imaging characteristics of the falciform ligament and the LTH pathologies is crucial for accurate diagnosis and management.

**Critical relevance statement:**

Recognition of the falciform ligament and the ligamentum teres hepatis pathologies is critical for accurate diagnosis, surgical planning, and avoiding misinterpretation of pseudolesions. Cross-sectional imaging helps identify pathologies of these structures, guide management, and avoid unnecessary interventions.

**Key Points:**

- The falciform ligament and the ligamentum teres hepatis have critical anatomical relationships that make them susceptible to various pathologies.
- Cross-sectional imaging is essential for the diagnosis of pathologies affecting these structures.
- Pseudolesions associated with these structures can mimic true liver pathology on CT and MRI.

**Graphical Abstract:**

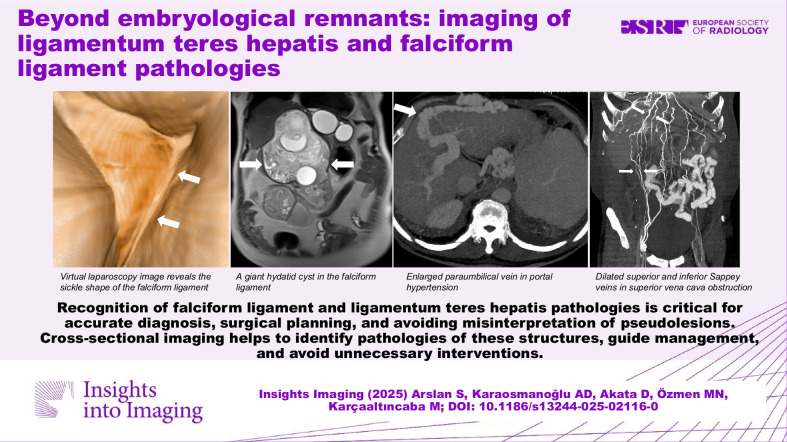

## Introduction

The falciform ligament (FL) and the ligamentum teres hepatis (LTH) have long been considered insignificant embryological remnants. However, the complex anatomical relationships of the FL and LTH with the liver, diaphragm, retroperitoneum, abdominal wall, and thoracic wall may lead to the involvement of these structures in various diseases. Cross-sectional imaging plays an essential role in evaluating diseases of the FL and LTH. In this review, we will discuss the normal anatomy and the role of cross-sectional imaging in evaluating the common and rare diseases affecting these structures.

## Anatomy

The FL, embryologically derived from the ventral mesentery, is a sickle-shaped, double-layered fold of the parietal peritoneum extending from the anterior abdominal wall to the liver. The FL divides the left liver lobe into segments 2–3 and segments 4 and attaches to the liver and the inferior aspect of the diaphragm. The FL also divides the subphrenic space into a left and a right side. The proximal portion of the FL diverges to intervene with the right and left coronary ligaments, which surround the bare liver area. The FL contains the LTH (or the round ligament of the liver), paraumbilical veins, and a variable amount of fat. The LTH, the fetal remnant of the umbilical vein that delivers oxygenated blood from the placenta to the left portal vein, is located in the free edge of the FL. The arterial supply of the FL arises from the middle hepatic artery and the left phrenic artery. Veins of the FL (paraumbilical veins- Burrow’s and Sappey’s-) establish anastomoses between the veins of the anterior abdominal wall and the portal vein, hypogastric, and iliac veins. Lymphatics drain into the superficial lymphatics of the liver [1, 2].

**Fig. 1:**
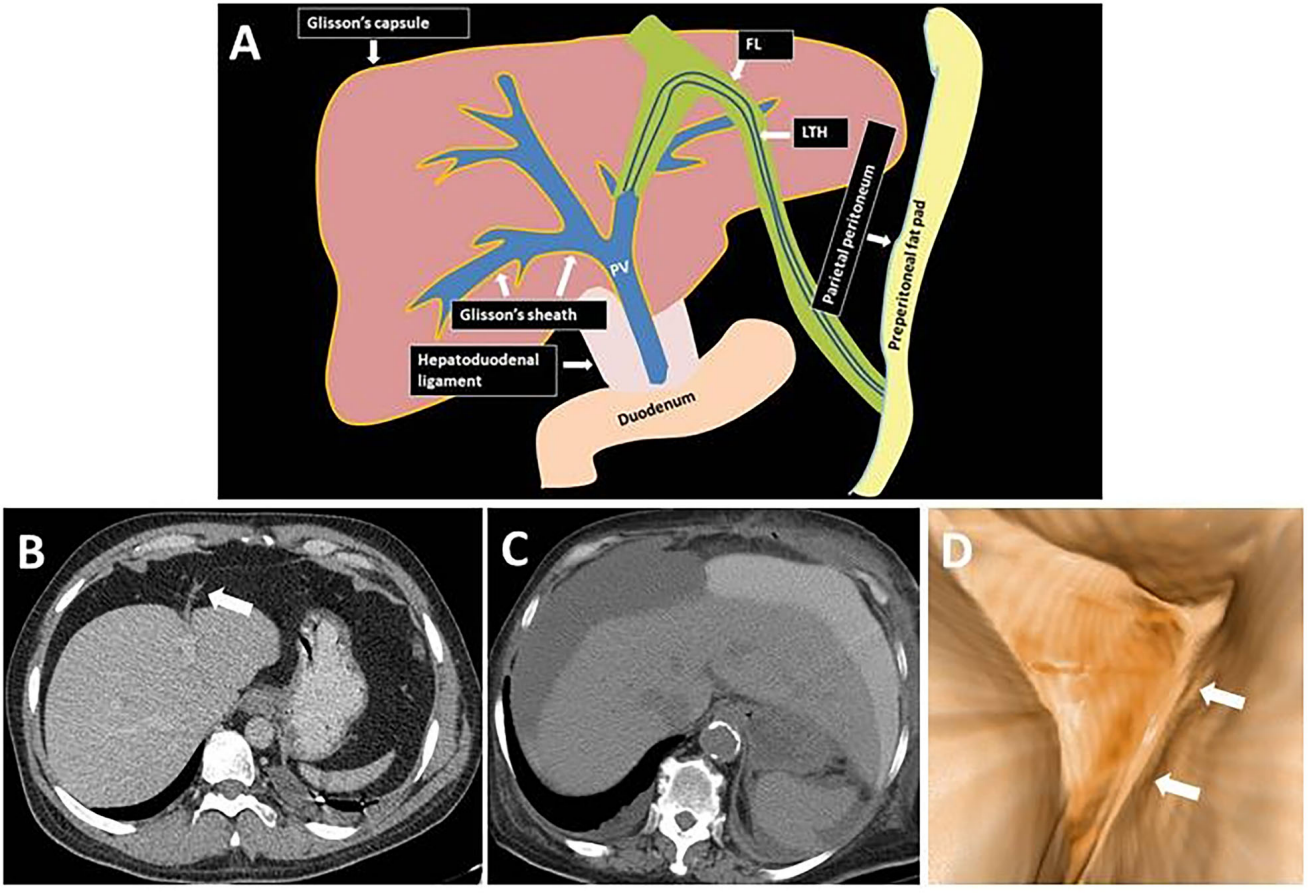
**A** Illustration depicting the anatomical relationships of the FL and LTH. Summary pathway: Retroperitoneum → Hepatoduodenal ligament → Glisson’s capsule/sheath → FL and LTH → Preperitoneal fat/anterior abdominal wall. **B** Axial plane contrast-enhanced CT image depicts the FL (arrow) extending obliquely from the left liver lobe to the anterior abdominal wall. **C** Axial plane CT image shows the right and left subphrenic spaces in a 72-year-old female patient with anastomotic leakage after small bowel resection. Note the oral contrast leakage into the left subphrenic space. The FL prevented the passage of oral contrast into the right subphrenic space. **D** Virtual laparoscopy image reveals the sickle shape of the FL. FL, falciform ligament; LTH, ligamentuım teres hepatis; PV, portal vein

The FL and LTH have complex anatomical relationships with the liver, diaphragm, retroperitoneum, abdominal wall, and thoracic wall. On the right, the retromesenteric plane, which is a part of the retroperitoneum, extends to the level of the right inferior coronary ligament and the bare area of the liver and communicates with the liver hilum through the subperitoneal space of the hepatoduodenal ligament. The subperitoneal space of the hepatoduodenal ligament continues into the Glisson’s sheath, which is defined as Glisson’s capsule surrounding the intrahepatic portion of the portal system. Glisson’s sheath also extends in the opposite direction into the LTH and FL. As the LTH travels within the FL, it reaches the umbilicus, where it connects with the preperitoneal fat layer of the anterior abdominal wall [1, 2] (Fig. 1A and Supplementary Video 1).

The FL is usually not visible on a plain abdominal X-ray unless pneumoperitoneum is present. On ultrasonography (US), the FL is visible as a round echogenic structure between the medial and lateral segments of the left liver lobe. On computed tomography (CT) and magnetic resonance imaging (MRI), the FL extends obliquely between the left liver lobe and the umbilicus in the anteroposterior plane [1] (Fig. 1B–D). Normal veins of the FL are sometimes visible on cross-sectional imaging. On cross-sectional imaging, it is easier to follow the course of the FL in the presence of intra-abdominal free fluid or air.

## Surgical considerations

The FL is an important anatomical landmark during hepatobiliary surgery. Hepatic veins, which are non-visible from the bare area of the liver, are located inferior to the FL. The LTH is a marker to identify the Rex recess, the remnant of the umbilical vein located between segments 3 and 4. The left portal vein, the target of the Meso-Rex bypass in extrahepatic portal vein thrombosis, is located in the Rex recess (Supplementary Material 1). The bile ducts of the left liver lobe are located above the left portal vein in the Rex recess, and the arteries are located below it [1–3].

Besides their importance as anatomical landmarks, the FL and LTH have been used in various surgical procedures. Surgical applications of the FL and LTH include but are not limited to coverage of gallbladder bed to reduce cholecystectomy complications, coverage of pancreas, liver, or spleen surfaces following injury, venous repair and reconstruction, bile duct repair and reconstruction, peptic ulcer repair, wrapping of bilioenteric / pancreaticojejunostomy stoma or duodenal stump, treatment of hiatal hernia and gastroesophageal reflux [4].

## Anatomical variations

**Fig. 2:**
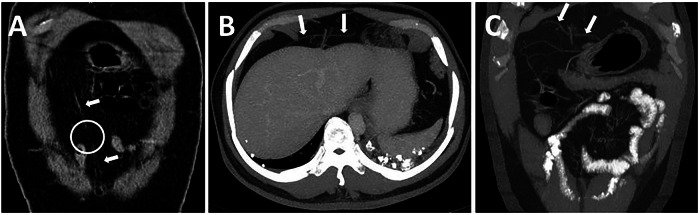
**A** 75-year-old male patient without a known surgical history underwent a CT scan to investigate epigastric pain. The coronal plane CT image shows a defect (circle) in the middle part of the FL (arrows). **B**, **C** FL defect in a 34-year-old male patient without a known surgical history. Axial (**B**) and coronal (**C**) plane contrast-enhanced CT images show an omental vein penetrating the FL, indicating the FL defect at this site (asymptomatic omental herniation through the FL)

There are several anatomical variations of the FL and LTH. A defect of the FL is usually a congenital defect, but it can also be caused by trauma. It may be found incidentally on imaging or cadaveric studies, but an internal hernia can develop from this defect [5]. On cross-sectional imaging, a vessel penetrating the FL is a finding of the defect in the FL (Fig. 2).

**Fig. 3:**
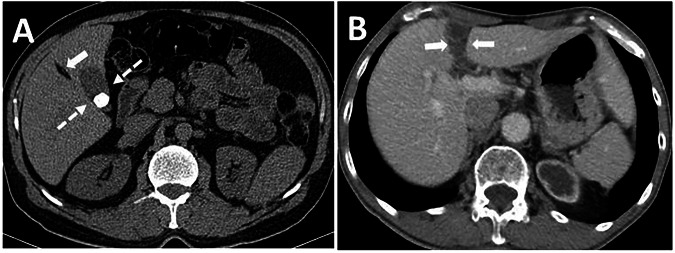
**A** The unenhanced axial plane CT image shows the right-sided LTH (arrow) and the gallbladder located on the left of the LTH (dashed arrows). Segment IV was atrophic (not shown). **B** Axial plane contrast-enhanced CT image shows the gallbladder (arrows), which is located in the FL

Right-sided LTH is another anatomical variation that may be associated with portal vein anomalies, biliary system anomalies, and segment IV atrophy. Right-sided LTH and associated anomalies are important in hepatobiliary surgery. In individuals with a right-sided LTH, the gallbladder fundus is always positioned to the left of or directly beneath the LTH. Rarely, the gallbladder may be located in the FL, which can lead to diagnostic confusion and surgical complications (Fig. 3) [6, 7].

## Diseases of the FL and LTH

### Air presence in the FL

Free intraperitoneal air can spread into the FL or outline the FL (Supplementary Material 2). Upright chest radiographs and lateral decubitus abdominal views are sensitive for detecting pneumoperitoneum; however, only supine radiographs are often feasible for critically ill patients. The FL or Silver sign is a useful indicator of pneumoperitoneum on supine abdominal radiographs. It reflects free intraperitoneal air outlining the FL and can aid diagnosis when upright imaging is not possible [8, 9].

### Infection, inflammation

**Fig. 4:**
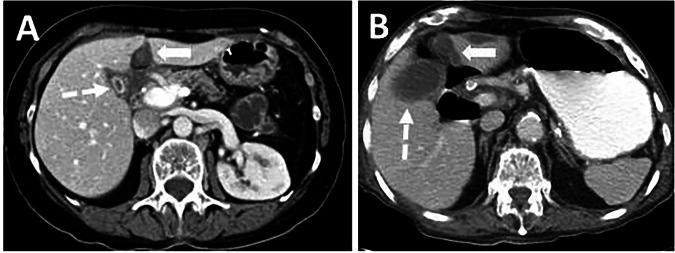
Two different patients with acute cholecystitis. **A** Axial plane CT image shows the wall thickening of the gallbladder and mucosal enhancement (dashed arrow). Inflammation is also observed in the FL and LTH (arrow). **B** Axial plane CT image shows the mildly distended gallbladder with wall thickening (dashed arrow). A fluid collection in the FL is also noted (arrow)

**Fig. 5:**
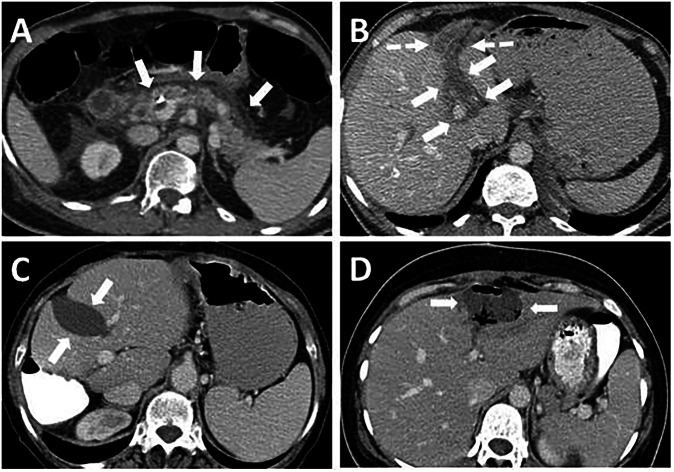
FL and LTH involvement in pancreatitis. **A**, **B** 68-year-old male patient presenting with epigastric pain. (A) Axial plane contrast-enhanced CT image shows peripancreatic fat stranding (arrows) compatible with acute pancreatitis. **B** Inflammations of the FL (dashed arrows) and LTH (arrows) are also present. **C** 50-year-old male patient with a history of right hepatectomy for metastatic colorectal cancer two years ago. He also had a history of acute pancreatitis 6 weeks ago and now presents with right upper quadrant pain. The axial plane CT image shows a fluid collection in the FL (arrows). The lesion was considered to be a pseudocyst. Follow-up imaging study demonstrated the decrease in the lesion size (not shown). **D** 68-year-old male patient with a history of acute pancreatitis and FL abscess. Axial plane contrast-enhanced CT image shows a fluid collection with air-fluid levels in the FL (arrows)

Due to the intimate relationship of the FL and LTH with the liver, gallbladder, porta hepatis, and retroperitoneum, infections and inflammatory conditions of these sites may affect the FL and LTH. Apart from this relationship, complex vascular and lymphatic pathways between the FL and the liver, diaphragm, retroperitoneum, abdominal wall, and thoracic wall may lead to the FL being affected by diseases of these regions (Figs. 4, 5 and Supplementary Material 3). The anatomical relationship between the retroperitoneum and the anterior abdominal wall, via the FL and LTH, may result in the Cullen sign. This is characterized by hemorrhagic discolouration of the umbilical area, caused by intraperitoneal hemorrhage from any cause. It is most commonly observed in patients with acute pancreatitis [1].

### Tumors and tumor-like conditions

**Fig. 6:**
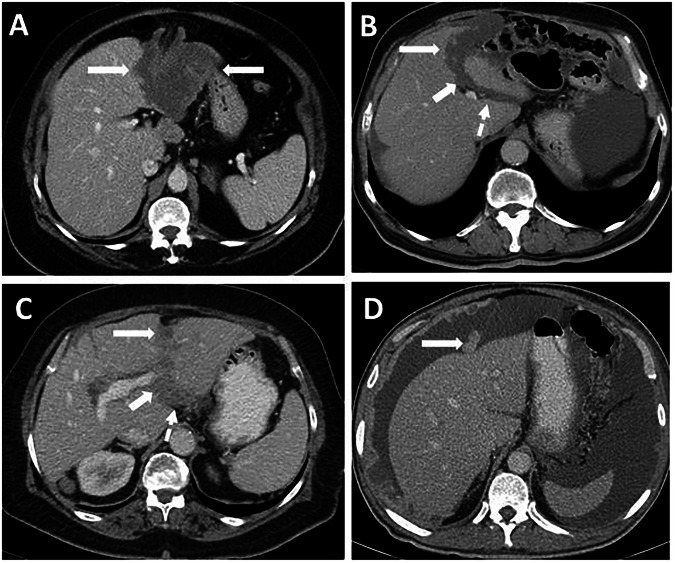
Secondary tumors of the FL and LTH. **A** 69-year-old female patient with cholangiocarcinoma. The mass (arrows) invades the FL and LTH and extends to both sides of these ligaments. **B**–**D** Secondary involvement of the FL and LTH from peritoneal carcinomatosis and sarcomatosis in three patients. FL (long arrows), LTH (short arrows), and hepatogastric ligament (dashed arrows) involvement in pseudomyxoma peritonei secondary to mucinous adenocarcinoma of the appendix (**B**) and peritoneal carcinomatosis secondary to high-grade serous carcinoma of the ovary (**C**). **D** FL (arrow) involvement in peritoneal sarcomatosis secondary to retroperitoneal liposarcoma

Various tumors and tumor-like conditions can develop in the FL and LTH, which may be asymptomatic or cause abdominal pain. The most common tumors of FL and LTH are secondary tumors arising from the liver and peritoneum (Fig. 6).

**Fig. 7:**
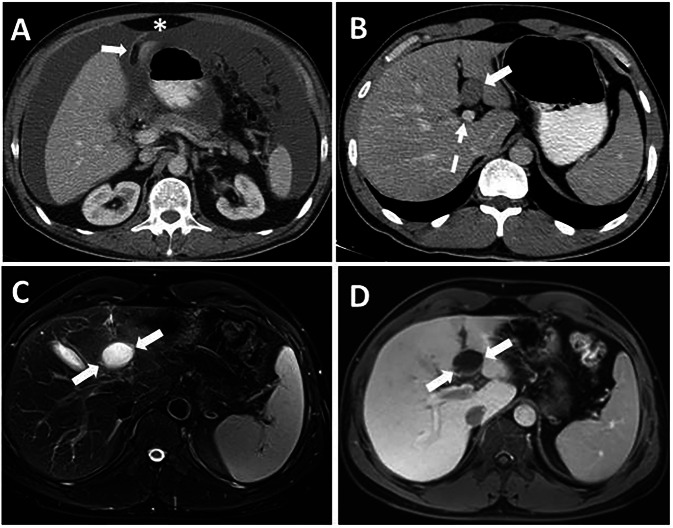
Primary tumors of the FL. **A** Lipoma of the FL (arrow). Note its association with FL, contrary to the preperitoneal fat pad (asterisk). **B**–**D** 37-year-old male patient with known gastric cancer underwent cross-sectional imaging for evaluation of distant metastases. **B** Axial plane contrast-enhanced CT image shows a round lesion (arrow) in the Rex recess anterior to the left portal vein (dashed arrow). Axial plane fat-saturated T2-weighted (**C**) and contrast-enhanced fat-saturated T1-weighted (**D**) MRI images denote that the lesion is a pure cystic lesion without a solid component. Histopathological findings after resection revealed a mesothelial cyst

**Fig. 8:**
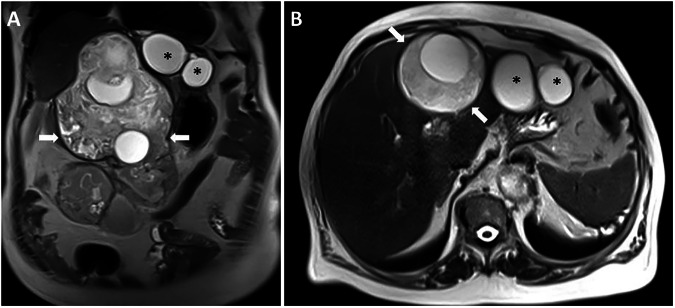
84-year-old female patient with epigastric pain underwent an abdominal MRI exam. Coronal (**A**) and axial (**B**) plane T2-weighted MRI images show a giant hydatid cyst (arrows) in the FL. Note small hydatid cysts (asterisks) in the left liver lobe

Primary tumors of the FL and LTH are extremely rare, with only a few case reports documented in the literature. Rare primary tumors of FL and LTH are lipoma, perivascular epithelioid cell tumor, fibroma, lymphangioma, gastrointestinal stromal tumor, paraganglioma, mature teratoma, gastrointestinal stromal tumor, clear cell myomelanocytic tumor, malignant mesothelioma and various types of sarcoma. Lipomas arise from adipocytes in the FL and can be distinguished from the preperitoneal fat pad by their association with the FL (Fig. 7A) [10–14]. Cystic lesions of the FL include congenital mesothelial or epithelial cysts, lymphangioma, and hydatid cysts, which have similar imaging features to those seen elsewhere in the body (Figs. 7, 8 and Supplementary Material 4) [15, 16]. In addition, lymphocele, abscess, bilioma, pseudocyst, and hemorrhage of surrounding structures may extend beyond the FL and cause a mass effect. Patient history is essential to differentiate these conditions.

Rarely, macroscopic mass-forming localized hepatic tuberculosis involves the FL and mimics malignant tumors owing to diffusion restriction and fluorodeoxyglucose avidity. On MRI, T2 hypointensity and ring-like contrast enhancement can be used to differentiate tuberculomas from neoplastic diseases; however, histopathological examination may be required for definitive diagnosis [17] (Supplementary Material 5).

### Calcification

**Fig. 9:**
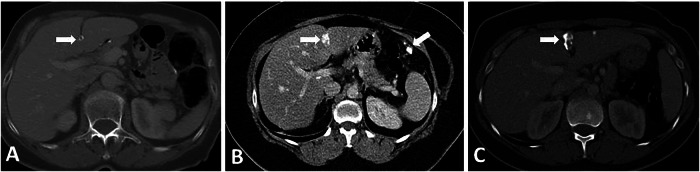
Calcified lesions of the FL. **A** 37-year-old female patient is presenting with epigastric pain. Axial plane contrast-enhanced CT image shows a small calcified lesion (arrow) in the FL, which may represent fat necrosis. **B** 42-year-old female patient with known ovarian cancer and peritoneal carcinomatosis. CT image demonstrates calcified implants in the FL and greater omentum (arrows). **C** 45-year-old male patient with recently diagnosed rectal cancer underwent a CT exam for evaluation of distant metastases. The CT image shows a calcified lesion in the FL. Histopathological confirmation after surgical extirpation revealed a hydatid cyst

Calcifications within the FL may be associated with fat necrosis [18]. However, it should be noted that some implants can be calcified in patients with peritoneal carcinomatosis, which may also affect the FL. Type 5 hydatid cyst may also be seen as a calcific lesion in the FL (Fig. 9).

### Paraumbilical veins-related diseases

In case of portal hypertension, paraumbilical veins may become enlarged to reduce portal vein pressure by shunting blood to the superior and inferior epigastric veins and may cause caput medusae sign, a network of dilated veins around the umbilicus (Supplementary Material 6) [19].

**Fig. 10:**
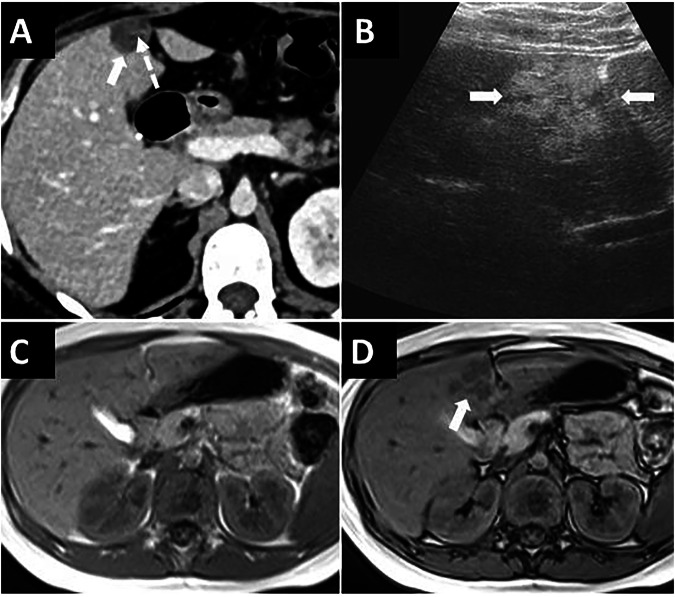
Focal fat near the FL. **A** 27-year-old male patient with a history of sleeve gastrectomy. Axial plane contrast-enhanced CT image shows focal fat (arrow) near the FL. Note the vessel traversing the lesion (dashed arrow). **B**–**D** 32-year-old female patient with known breast cancer. **B** Ultrasound image shows a hyperechoic lobulated lesion close to the FL (arrows). In-phase (**C**) and out-of-phase T1-weighted (**D**) MRI images confirm the lesion as a focal fat by the presence of a focal signal drop (arrow in **D**) on the out-of-phase image

The third inflow into the liver from the paraumbilical veins is thought to be the cause of hepatic pseudolesions near the FL, including focal fat (FF), focal fat sparing (FFS), and perfusion abnormalities. Heterogeneous fat accumulation in the liver may lead to diagnostic confusion [20]. The findings of FF or FFS may mimic metastasis on CT. However, typical sites of FF or FFS and vessels traversing the areas of abnormal attenuation can aid in the correct diagnosis. FF or FFS is typically seen in periportal distribution and around the FL. In atypical sites, such as abnormal left gastric vein-associated pseudolesions in segments 2 and 3, MRI can differentiate between metastasis and pseudolesion. On MRI, in- and out-of-phase T1-weighted imaging demonstrates focal signal drop on out-of-phase images in the presence of FF and focal lack of signal drop on out-phase images in the presence of FFS (Fig. 10) [21, 22].

Obstruction of the superior vena cava leads to multiple collaterals in the chest and abdominal wall, some of which result in portosystemic venous shunting via the paraumbilical veins. On contrast-enhanced images, increasing intrahepatic blood flow produces a focal wedge-shaped hyperdense area in segment 4, mimicking a hypervascular liver mass (Supplementary Material 7). The presence of chest and abdominal wall collaterals may raise the suspicion of superior vena cava obstruction, and the diagnosis should be confirmed by contrast-enhanced chest CT [21, 23].

**Fig. 11:**
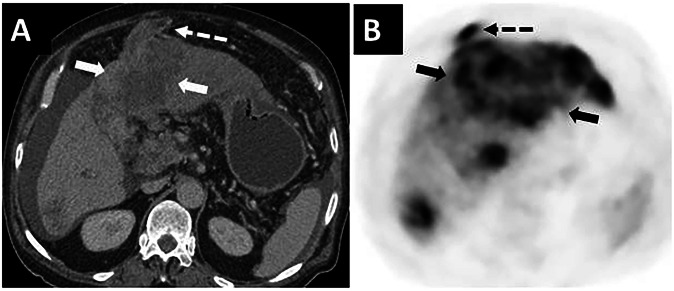
Tumor thrombus in the paraumbilical vein in a patient with known hepatocellular carcinoma. **A** Axial plane contrast-enhanced CT image and **B** choline PET-CT image show a diffuse infiltrating lesion in the left liver lobe (arrows) invading the left portal vein. Tumor thrombus in the paraumbilical vein is also noted (dashed arrows)

Hepatocellular carcinoma, the most common primary malignant tumor of the liver, tends to invade large veins. Tumor thrombus in the portal veins is found in up to 64% of patients, but tumor thrombus in the paraumbilical veins is extremely rare (Fig. 11) [24, 25].

### Other vascular diseases

**Fig. 12:**
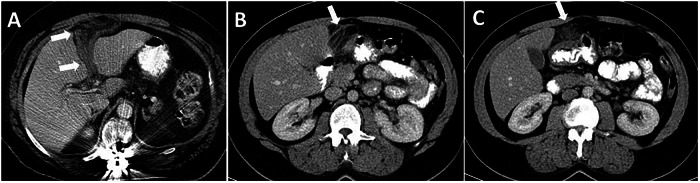
Torsion of the FL in two patients presenting with acute right upper quadrant pain. **A** Axial plane CT image of a 60-year-old female patient shows a mass-like area in the fissure of the FL with fat attenuation (arrows). **B**, **C** Axial plane consecutive CT images of a 35-year-old male patient show fat stranding along the course of the FL (arrows)

Acute torsion of the FL resulting in focal fat infarction is a rare cause of acute abdominal pain. Torsion of the FL has a similar pathological process to other well-known focal fat infarctions in the abdomen, such as omental infarction or epiploic appendagitis. Patients usually present with right upper quadrant pain. Blood tests can show mild elevation of inflammatory markers. As the differential diagnosis of right upper quadrant pain is broad, imaging plays a crucial role in the diagnosis. In the US, the increased fat echogenicity in the right upper quadrant or non-compressible heterogeneous hyperechoic mass may be seen, but it is non-specific, as many other inflammatory processes may cause it. On the next step, CT is preferable owing to the fast acquisition time in emergency patients. CT can show a well-circumscribed mass-like area in the fissure of the FL with fat attenuation and associated inflammatory changes in the adjacent fat planes (Fig. 12) [26, 27].

## Conclusion

The FL and LTH, traditionally considered embryological remnants, play a more critical role in clinical practice than previously recognized. Their complex anatomical relationships with surrounding structures make them susceptible to various pathological conditions. Many pathologies associated with FL and LTH may be encountered in radiological practice, especially inflammatory processes, tumors, and tumor-like lesions. Cross-sectional imaging is essential to diagnose these conditions accurately and to differentiate true pathologies from pseudolesions. A comprehensive understanding of the imaging features and anatomical relationships of these ligaments is essential for early detection, effective management, and the prevention of surgical complications.

## Supplementary information

ELECTRONIC SUPPLEMENTARY MATERIAL

Supplementary video 1R3

## Abbreviations

CT: Computed tomography
FF: Focal fat
FFS: Focal fat sparing
FL: Falciform ligament
LTH: Ligamentum teres hepatis
MRI: Magnetic resonance imaging
