# Supplementary material for: Beyond embryological remnants: imaging of ligamentum teres hepatis and falciform ligament pathologies
Source: Insights Imaging. 2025 Oct 25;16:227. doi: 10.1186/s13244-025-02116-0 (PMC12553636; doi:10.1186/s13244-025-02116-0)
Supplement: Supplementary file 1 — ELECTRONIC SUPPLEMENTARY MATERIAL [file 13244_2025_2116_MOESM1_ESM.pdf]

Beyond Embryological Remnants: Imaging of Ligamentum Teres Hepatis and Falciform Ligament Pathologies

ELECTRONIC SUPPLEMENTARY MATERIAL

Insights Imaging (2025) Arslan S, Karaosmanoğlu AD, Akata D, Özmen MN, Karçaaltıncaba M.

Supplementary video 1. Axial plane contiguous CT images of a patient with massive pneumoperitoneum clearly illustrate the entire course of the FL (arrows) from its attachment to the diaphragm and to the attachment site to the umbilicus.

Supplementary Figure 1:

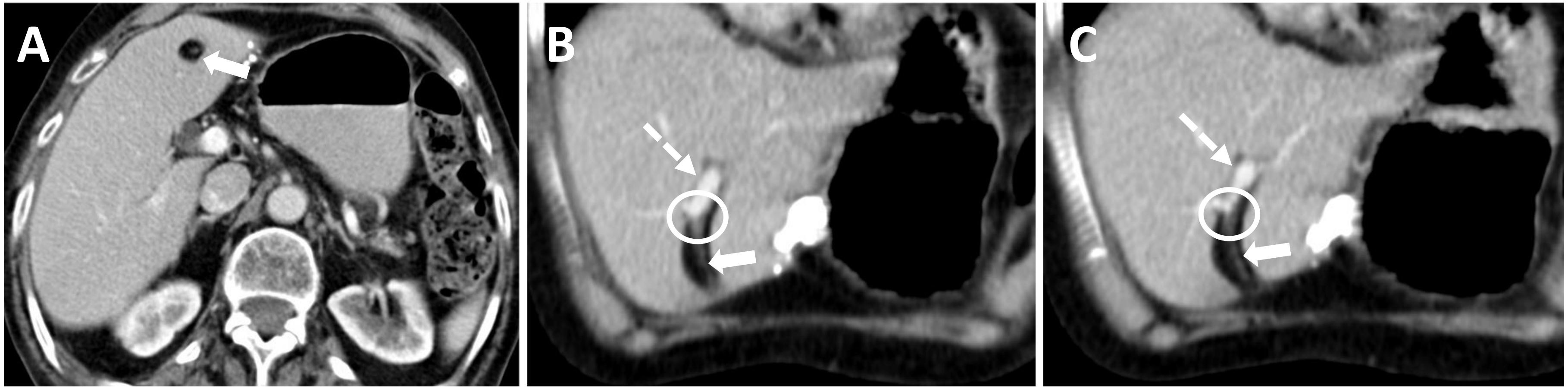

Axial and coronal plane contrast-enhanced CT images depict the LTH in the free edge of the FL (arrows in A-C), left portal vein (dashed arrows in B, C), and Rex recess (circles in B, C).

Supplementary Figure 2:

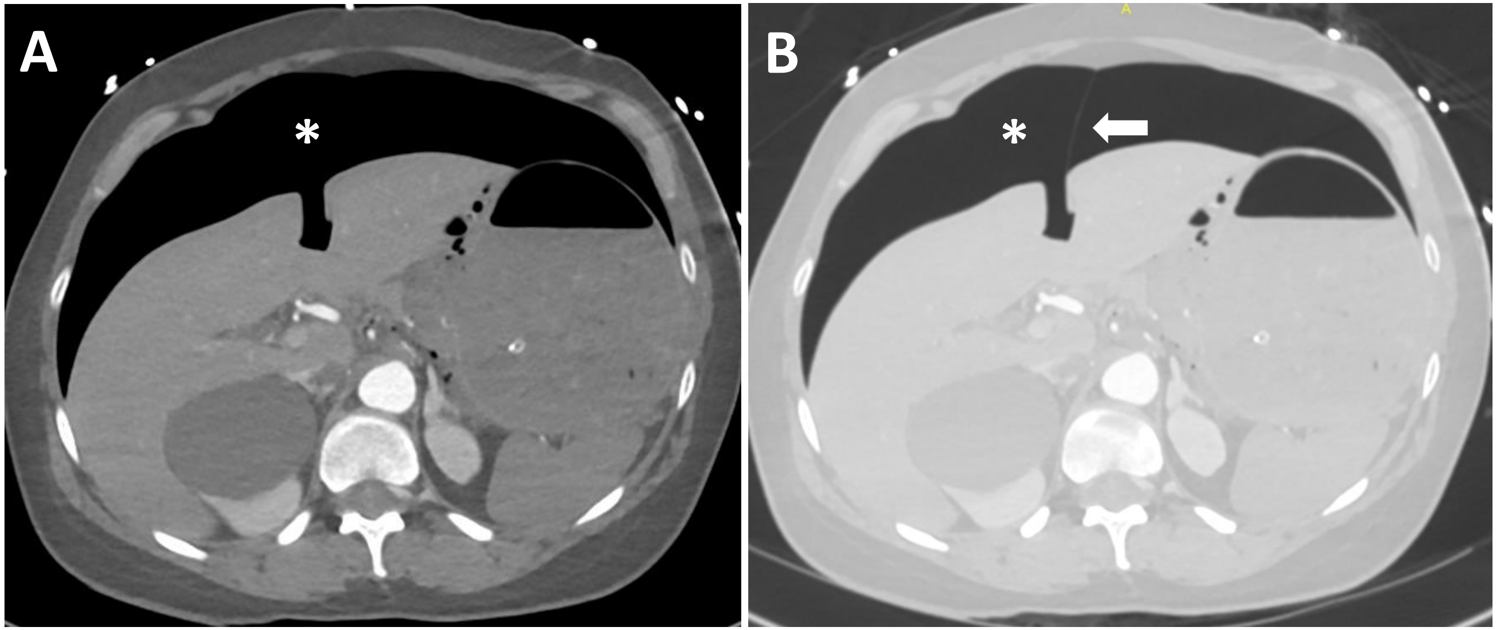

69-year-old female patient with left-sided colonic diverticulosis was referred to CT for evaluation of intra-abdominal free air after iatrogenic perforation of her sigmoid colon during colonoscopy. (A-B) The axial plane CT images depicts a massive pneumoperitoneum (asterisk) outlining the FL (arrow).

Supplementary Figure 3:

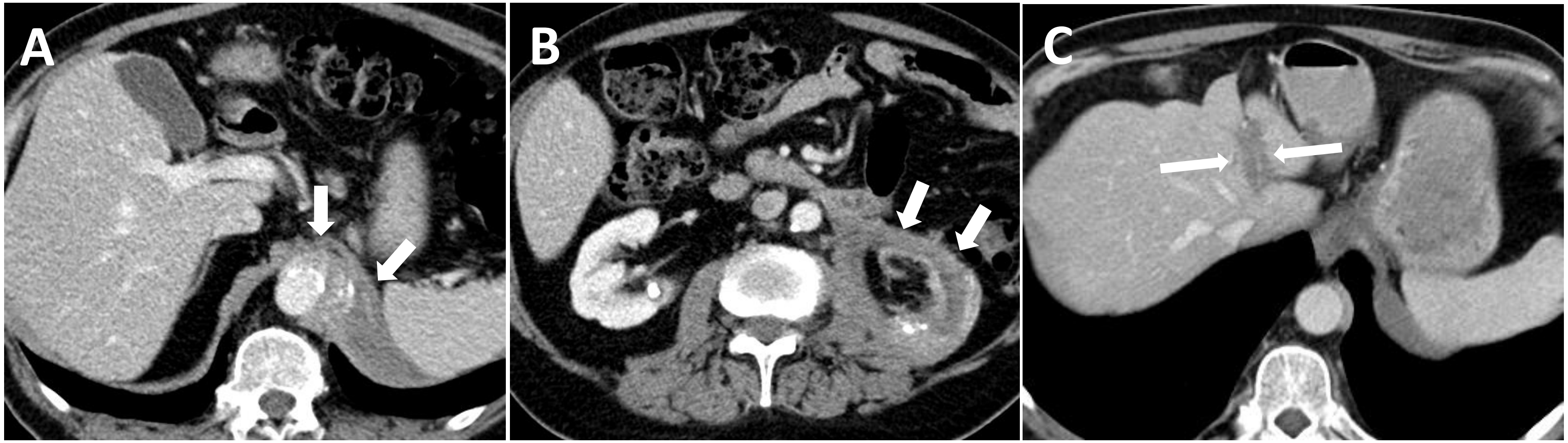

Involvement of the FL and LTH from IgG4-related disease. (A, B) Axial plane contrast-enhanced CT images show a soft tissue lesion in the left retroperitoneum encircling the aorta and atrophic left kidney (short arrows). (C) The section through the liver shows thickening of the FL and LTH (long arrows). Histopathological findings after the biopsy of the retroperitoneal mass revealed IgG4-related disease.

Supplementary Figure 4:

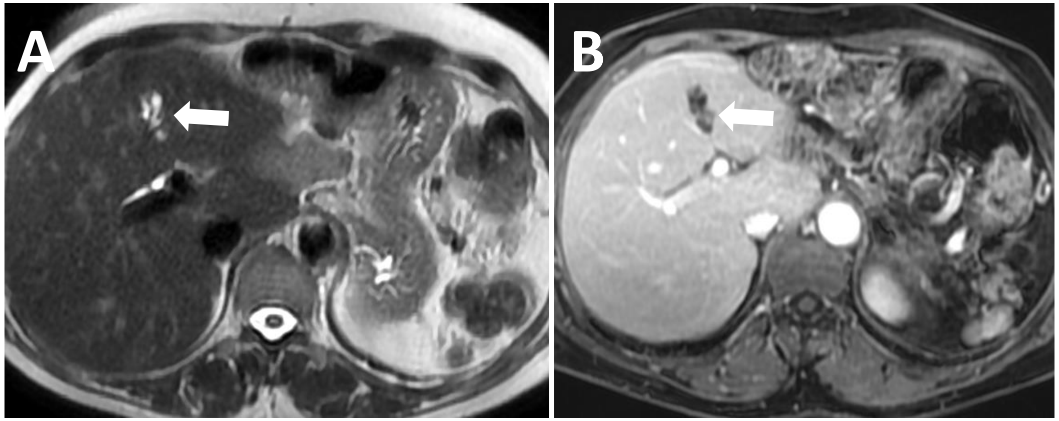

48-year-old male patient with recently diagnosed rectal adenocarcinoma underwent an abdominal MRI exam. Axial plane T2-weighted (A) and contrast-enhanced T1-weighted (B) images show a septated cystic lesion in the FL (arrow). The lesion was found to be representing a lymphangioma. Follow-up studies confirmed the stability of the lesion.

Supplementary Figure 5:

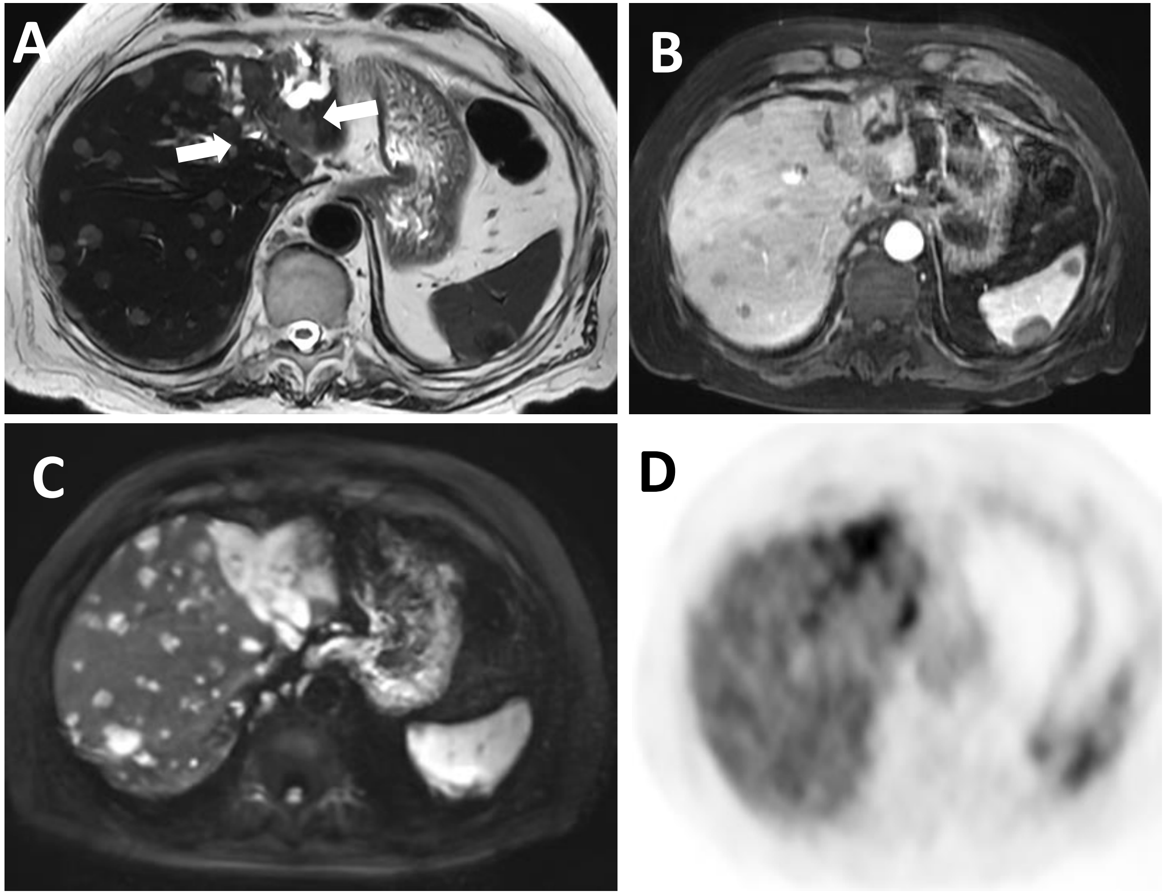

70-year-old male patient with known head and neck squamous cell carcinoma. Axial plane T2-weighted (A) and contrast-enhanced fat-saturated T1-weighted (B) MRI images show a mass invading the FL and causing dilatation of the biliary tree. There are also numerous masses in the liver and spleen. (C) High b-value diffusion-weighted MRI image shows the restricted diffusion of the masses. (D) The masses show high 18-fluorodeoxyglucose uptake on PET-CT images. The imaging features were suggestive of metastases; however, histopathological findings after trucut biopsy revealed tuberculosis.

Supplementary Figure 6:

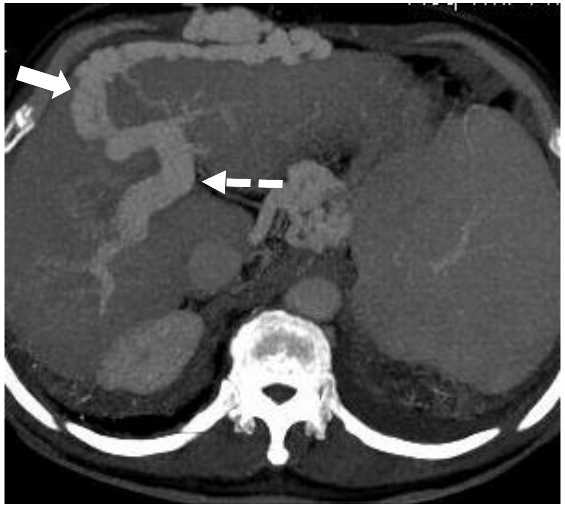

Axial plane contrast-enhanced maximum intensity projection CT image shows an enlarged paraumbilical vein (arrow) that drains the left portal vein (dashed arrow) in a 58-year-old male patient with a history of chronic liver disease and portal hypertension.

Supplementary Figure 7:

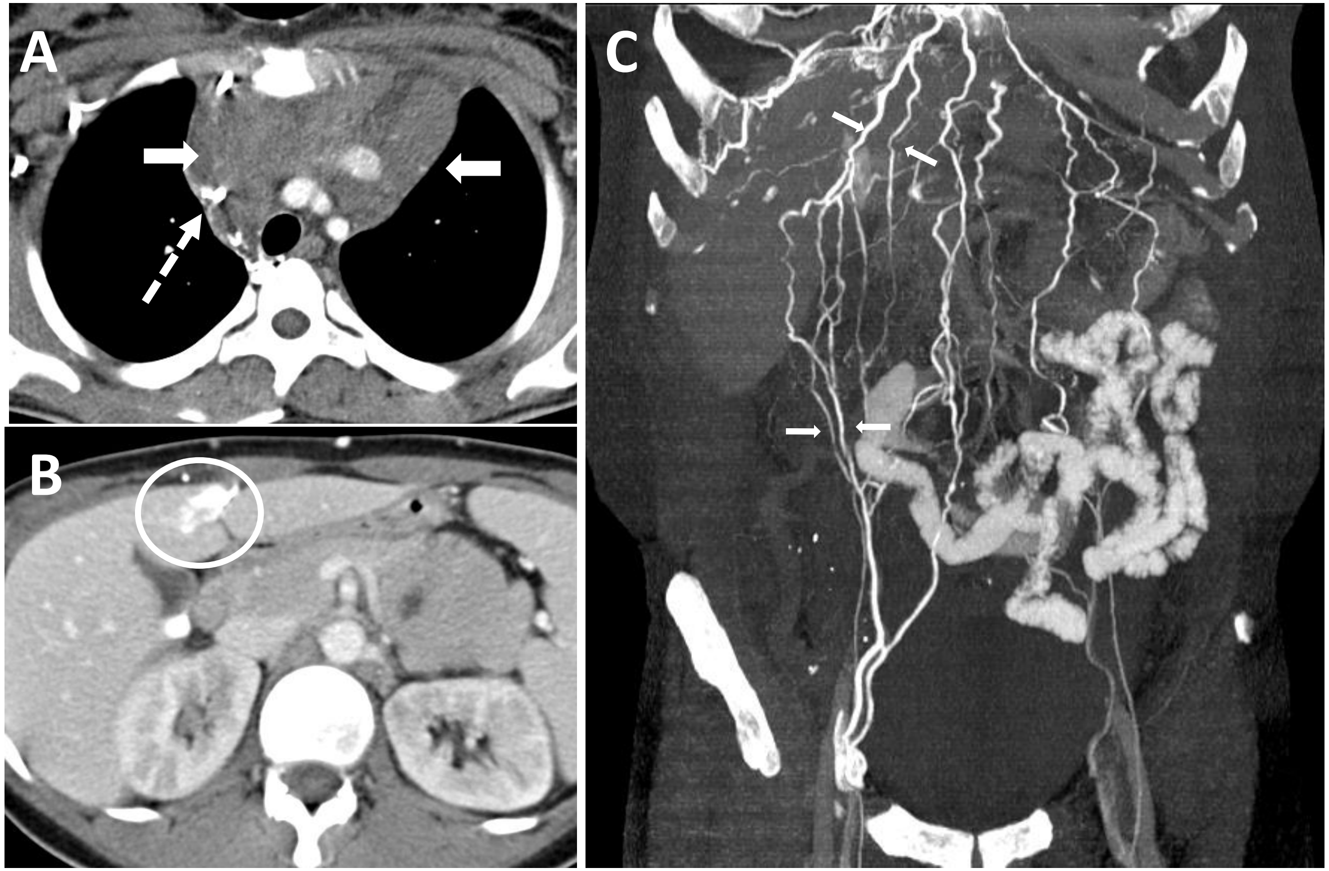

Hepatic pseudolesion secondary to the obstruction of the superior vena cava in a 20-year-old female patient with recently diagnosed lymphoma. (A) Axial plane contrast-enhanced chest CT image shows a large mediastinal mass (arrows) narrowing the superior vena cava (dashed arrow). (B) Axial plane contrast-enhanced abdominal CT image points to a focal wedge-shaped hyperdense area in segment 4 (circle) due to the portosystemic venous shunt. (C) Coronal oblique maximum intensity projection image shows the dilated superior and inferior Sappey veins (arrows) and multiple collateral veins in the abdominal wall.
